# Miscarriage Australia: the use of a human centered design approach to design and develop a website for those affected by miscarriage

**DOI:** 10.3389/fpubh.2023.1128768

**Published:** 2023-05-12

**Authors:** Jade Bilardi, Amy Webb, Van-Hau Trieu, Gemma Sharp, Jennifer McIntosh, Meredith Temple-Smith

**Affiliations:** ^1^Central Clinical School, Monash University, Melbourne, VIC, Australia; ^2^Department of General Practice, University of Melbourne, Melbourne, VIC, Australia; ^3^Melbourne Sexual Health Centre, Alfred Health, Melbourne, VIC, Australia; ^4^Department of Information Systems and Business Analytics, Deakin University, Melbourne, VIC, Australia; ^5^Department of Neuroscience, Monash University, Melbourne, VIC, Australia; ^6^HumaniSE Lab, Faculty of Information Technology, Monash University, Melbourne, VIC, Australia; ^7^School of Population and Global Health, The University of Melbourne, Melbourne, VIC, Australia

**Keywords:** miscarriage, pregnancy loss experience, digital health (eHealth), human centered design (HCD), psychological support, early pregnancy loss

## Abstract

**Background:**

Past research has shown that Australians affected by miscarriage want a website specific to both miscarriage and their local region that is accessible, comprehensive, evidence-based and informed by experts. The aim of this study was to design, develop and evaluate the Miscarriage Australia website using human centered design.

**Methods:**

A four stage human centered design approach was used to develop the Miscarriage Australia website which aimed to: (1) *Understand* the issue and why users need a website; (2) *Define* users’ specific needs; (3) *Design* solutions to meet those needs; and (4) *Evaluate* the design by testing with end users. Across the four stages, various types of data and data analysis were developed and utilized including interviews, desktop research, development of personas and tone of voice, followed by usability testing. Process and content were guided by designers, developers and an expert advisory committee of key stakeholders.

**Results:**

Analysis and synthesis of user research across Stages 1 and Stage 2 found 11 key themes pertaining to user’s miscarriage experiences and support needs. Using the themes, common experiences, goals, motivations and behaviors of users were identified and similar user types grouped and used to inform the development of two personas. Using the personas and user research findings, design elements (Stage 3) including the “tone of voice guidelines” were developed recommending the Miscarriage Australia website be calm, empathetic, hopeful and authoritative. The tone of voice guidelines guided branding and over 100 pages of content was informed by the research team and reviewed by a 13-member Expert Clinical Advisory Committee over two rounds to ensure it was evidence based and reflected best practice. Using a contextual inquiry approach, usability testing was undertaken with 8 end users to test a low fidelity mockup and high-fidelity prototype of the website. Overall, end users reported the website was highly acceptable in terms of the design, content, layout, language and terminology, describing it in line with the intended tone of voice. Users reported the website was easy to use and navigate and provided useful and appropriate content and resources. Minor areas for improvement included slight changes to specific images, improved links for navigating sections, and a title change to one section heading.

**Conclusion:**

The Miscarriage Australia website was successfully implemented and commended by users as meeting their needs. As a result of using human centered design, the Miscarriage Australia website provides an ideal template or blueprint on how to develop a successful and useful digital resource for users, particularly around sensitive women’s health issues.

## Introduction

In Australia, miscarriage is defined as the loss of a pregnancy before 20 weeks gestation (1). Miscarriage is estimated to occur in approximately 20–25% of all confirmed pregnancies (2–4) and while the physical aspects are generally managed easily medically, it often results in significant psychosocial sequelae (5–9).

Despite the frequency with which miscarriage occurs and the significant psychosocial sequelae, adequate support from healthcare providers and social networks at the time of miscarriage is all too often lacking (10–22). The lack of support is highly concerning, as it is a major risk factor for psychological morbidity following miscarriage (8, 17). Evidence consistently shows positive support experiences can buffer the loss and lead to better psychosocial outcomes (23, 24), including reducing the risk of long-term psychological morbidities and suicide (25).

An increasingly common format for providing psychosocial support and health information is through eHealth or digital health, with the number of users accessing the internet for health services more than doubling in the last decade in Australia (26). Research has shown that people affected by miscarriage often seek information and support online when faced with a lack of support or insufficient information at the time of miscarriage as it is anonymous, easy to access and fills a gap in support needs (27–30). Our research group has found that individuals often struggle to find a singular reputable Australian based miscarriage website that is accessible, comprehensive, evidence-based and informed by miscarriage experts and healthcare providers (11, 30, 31).

Since the information and support desired by those affected by miscarriage is often not routinely offered or provided in the manner they need, a wider range of support options is required. Web-based services offer confidential, anonymous, and convenient access to internet users and is accessible by the geographically isolated. In Australia, almost everyone has access to the internet (99%), with 91% of Australians having a home connection (32) and the most commonly used device to access the internet being a mobile phone (32). Past research has found that web-based services are effective in delivering information, meeting the psychosocial needs of vulnerable groups (33), and supporting people to overcome mental health difficulties (34, 35).

The aim of this study was to design, develop and evaluate the Miscarriage Australia website using a human centered design (HCD) approach to increase accessible resources to support those affected by miscarriage.

### Human centered design

Human centered design (HCD) was used to develop the Miscarriage Australia website as the method offers an effective solution to address complex health care challenges (36). HCD is considered best practice for design and software development to meet diverse end user’s needs in an accessible way (37).

The International Organization for Standardization (ISO) provides best practice standards related to human centered design (38). The ISO standard 9241–210: 2019 defines human centered design as an *“interactive systems development that aims to make systems usable and useful by focusing on the users, their needs and requirements, and by applying human factors/ergonomics, and usability knowledge and techniques. This approach enhances effectiveness and efficiency, improves human well-being, user satisfaction, accessibility and sustainability …*” (38). Ultimately, HCD aims to involve users throughout the design process to ensure the final product or service is tailor-made to meet user needs (39).

The six principles of HCD are:

1. The design is based upon an explicit understanding of users, tasks and environment
2. Users are involved throughout design and development
3. The design is driven and refined by user-centered evaluation
4. The process is iterative
5. The design addresses the whole user experience
6. The design team includes multidisciplinary skills and perspectives (38).

**Figure 1:**
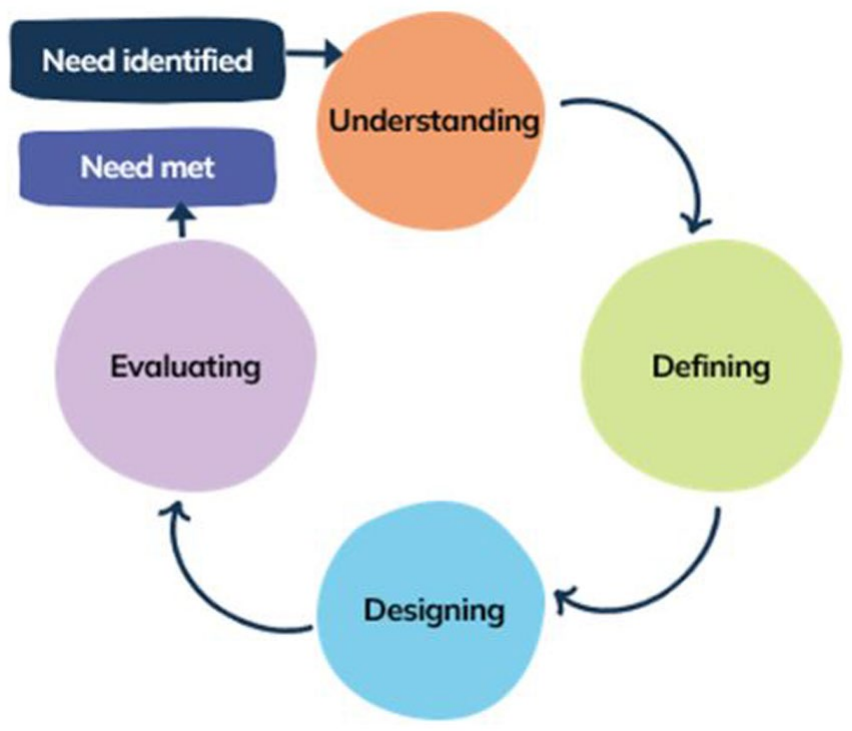
Human centered design process based on four main stages as outlined by ISO 9241-210:2019.

The four main stages in the process are to: (see Figure 1).

1. *Understand* and specify the context of use.

2. *Define* the user requirements.

3. *Design* solutions to meet the requirements.

4. *Evaluate* the design against requirements (40).

The key strength of human centered design is the active involvement of end users, beginning at the start of the design process (41). When referring to human centered design, the ISO noted in their updated 2019 standard that HCD *“addresses impacts on a number of stakeholders, not just those typically considered as users”* (38). In the case of the Miscarriage Australia website, it was important not to just consider women affected by miscarriage but also other stakeholders including healthcare providers.

## Materials and methods

### Ethics statement

Ethics approval for this study was granted on the 16th November 2020 by the Monash University Human Research Ethics Committee, Victoria, Australia (Ethics Number 25757).

### Study design

#### Key stakeholders and multidisciplinary design team

In this project, users/key stakeholders included those affected by miscarriage and the healthcare providers caring for them. Healthcare providers were represented by an expert clinical advisory committee of 13 medical and allied healthcare professionals working in primary care, obstetrics and gynecology, midwifery, clinical psychology, women’s mental health, perinatal mental health, bereavement care, *in vitro* fertilization (IVF) and infertility. The role of the Committee was to review content relevant to their area of expertise to ensure the information and resources were medically accurate, evidence based and reflected current best practice in their field.

Our multidisciplinary design team included our research team which comprised multidisciplinary researchers with expertise in social research and sexual and reproductive health (JB, MTS, JM, GS, JM), primary care (MTS, JM, JB), allied health (GS), eHealth (JM, VT) and IT (VT) and two specialist HCD companies, Splendid Studio (hereafter referred to as designers) who assisted with the user research, persona development and tone of voice guidelines (Stages 1 and 2) and Sixheads (hereafter referred to as developers) who assisted with branding and website development and testing (Stages 3 and 4).

The four main stages of the HCD approach as shown in Figure 1 were undertaken across the project.

### Stage 1: Understand

The key strength of human centered design is the active involvement and engagement of end users, beginning at the start of the design process and allowing for a clear understanding at the outset of users’ experiences, needs and requirements (41). The vital first step in any design project is to undertake user research to ensure the users’ needs are captured (42).

#### Previous study data

Prior to this study, the research team had undertaken numerous pilot studies with women (*n* = 28) and male partners (*n* = 16) living in Australian and affected by miscarriage. Those affected by miscarriage had been involved in semi-structured interviews about their experience of miscarriage, their support needs, and health seeking behaviors, including online support. These data have been published elsewhere (11, 14, 30, 31, 43). Key findings from three of these studies (11, 30, 31), related to online information and support needs, were collated and tabled by JB and MTS and provided to the designers along with the full published papers.

#### Desktop research

Along with interview data, the designers also undertook their own desktop research which involved auditing and cataloging current online content and best practice examples of similar pregnancy loss projects worldwide to compare it against needs identified in the user research data.

### Stage 2: Define

The define stage aims to make sense of the problem from the user’s perspective and identify users’ needs. This stage involved (1) affinity mapping of user research and (2) the development of user personas.

#### Affinity mapping of user research

In addition to the collated previous study findings, 10 de-identified interview transcripts with eight women and two male partners from a recent study around the support needs of those affected by miscarriage (31) were also provided to the designers. Interview transcripts were from individuals who resided in Australia, had a good understanding of English, had been affected by miscarriage as women, partners or family members more than 3 months but less than 2 years ago. Transcripts were chosen purposively by the team members for breadth and depth of participant experience and support needs. Two men were included in the 10 chosen manuscripts to ensure partners’ voices and experiences were also considered. The sample included a range of participants by age, gender, geography, number of miscarriages and number of living children (see Results section).

Affinity mapping was undertaken to analyze and synthesize the interview data to define users’ needs and inform the development of representative personas. Less structured, yet similar to thematic analysis, affinity mapping allows designers to find patterns in the data across multiple data sets, helping them make sense of the relationships between concepts and ideas by organizing them into groups or categories (42). Post-it-notes were used to record individual insights or observations, which were then grouped into emerging themes or categories, the end result being a visual representation from the user’s perspective of their journey, steps in their journey, emotions, communication and the various stakeholders involved (42).

The designers started the affinity mapping process by firstly familiarizing themselves with the data sets provided by the research team. While further interview transcripts were available, the designers reported there was sufficient breadth, depth and meaning in the data provided to reach data saturation. While reviewing the interview transcripts and recordings, the designers noted their research observations or ideas, each on a single post-it-note. The post-it-notes were then sorted and organized into emerging themes or categories, firstly on a large wall and then in later ideations using MIRO software, an online whiteboard which digitally displayed all the post-it-note ideas. At this point, the designers met with the wider research team to discuss the groupings and emerging themes, further reviewing and refining the themes based on the team’s feedback and meeting one final time with the research team to agree on final themes. The final synthesis of research findings subsequently informed the design process (Stage 3). Affinity mapping findings are discussed further below in the Results section.

#### Persona development

Using the synthesized findings, the designers then developed two personas to represent end users. Personas are representations or archetypes of the typical users or target audience for the product or—in this case, users of the Miscarriage Australia website (44, 45). Personas generally include a name, personality, picture, scenario of use, user motivations, goals and behaviors (41, 45). Informed through the synthesis of research data, personification of data into this format is extremely effective in driving design outcomes. They allow the design to be examined against the needs of different personas (41). They also helped inform the desired look, feel, design and content needs of the website from a user perspective. The designers liaised regularly with the research team in the development of the personas to ensure consensus was reached on the personas and thus typical users’ behaviors, motivations and goals in using the website.

### Stage 3: Design

Once the user requirements were established and the personas developed, the design process began. Stage 3 involved (a) the development of tone of voice guidelines, (b) branding, and (c) content creation.

#### Tone of voice guidelines

The personas and user research findings were used to inform the website design, which included developing tone of voice guidelines, branding and content creation. According to Delin et al. (46) *“this means that the ‘look and feel’ of the brand is increasingly accompanied by branded language—in the commercial world, this is often termed ‘Tone of Voice’ (although it applies equally to written as well as spoken language). Branded language is deliberately developed so that it expresses the brand identity”* (p. 28). While a brand includes a logo and/or a name, it also reflects the personality, identity and values of the organization or company to their intended audience, and this is conveyed not just visually but also verbally (34, 46). The tone of voice guidelines anchored the content creation and also informed the branding colors, imagery and language used across the website.

#### Branding

Using the tone of voice guidelines as a guide, the developers began the branding process. The first step in developing Miscarriage Australia’s brand identity and design direction was for the developers to conduct a two-hour Discovery Workshop with the research team. Numerous activities aided decision making on the Miscarriage Australia brand purpose (why do we exist?), values (who we are?), audience (who we are talking to?) and identity or personality (what do we look like?). Following the Discovery Workshop, branding elements, including logo options, color palettes, typography, icons and imagery were drafted for the research team to review and further refined following two rounds of feedback from the team.

#### Content creation

Based on the user research data, the research team worked with the designers to table content needs and compile a site map to guide content creation. JB and AW wrote the main sections of content, with assistance from MTS initially and then the wider research team. Once a full first draft of the content was complete, the designers began the editing process to ensure the language and content met the needs of the personas and tone of voice guidelines. The designers liaised regularly with the research team throughout the copy-editing process to ensure editing changes did not impact text meaning or accuracy.

The expert advisory committee reviewed sections of copy relevant to their area of expertise. The copy underwent two rounds of review through the expert advisory committee while content was developed and finalized.

### Stage 4: Evaluate

Once the design and content needs were established, the developers began the build and testing of the website.

#### Usability testing

Usability testing involves testing a product or service with a sample of intended end-users to gain insights to allow optimization of design and user experience (47). The optimal sample size for testing to discover problems is a contentious and highly debated issue in the field (48, 49). Studies by Nielsen, a pioneer in this area whose advice is commonly cited, showed that testing with five users from any user group will elicit 80% of interface usability problems (48, 50). According to Alroobae and Mayhew’s study findings, the appropriate sample size needed for a variety of study purposes, to find a few major issues and mainly minor issues largely around layout and formatting, is eight end users (48). According to Macefield (49) *“there is no ‘one size fits all’ figure for the optimal group size for usability studies related to problem discovery. Rather, this should be influenced by the study’s context and complexity”* (p. 38). The Miscarriage Australia website was intended to be an informational website which did not require ecommerce or complex functions. The purpose of usability testing was primarily to test the visual elements and content of the website including alignment with the tone of voice guidelines, and the website’s acceptability, usability and functionality. The developers advised two cycles of iterative testing would be sufficient during the development and testing stage. Usability testing was undertaken early in the design phase at low fidelity or rough mockup stage and then later at the high-fidelity or fully functional prototype stage. Low fidelity mock-up stage is where a rough sketch or framework of the structure and layout of the website is provided along with the visual design aspects including colors, images, logos and typography giving it a more realistic feel or look for what the final product will look like (44, 50). High fidelity prototype stage is when a fully functional mock-up is developed that acts like the final website and can be tested to ensure the actual coded site is functional and that the mock up has been brought to life successfully (44, 50).

At both these stages, contextual inquiry, a less formal, more cost-effective approach in testing, was undertaken whereby the developers observed and interviewed users as they viewed the low fidelity mockups or navigated through the high-fidelity prototype (44). They used one of the main methods in contextual inquiry [or participatory inquiry (41)], the “think aloud” method whereby participants were instructed to “think aloud” any thoughts they had as they navigated through each page of the website (51, 52).

##### Recruitment

To be eligible to participate in the usability testing, participants had to reside in Australia, have been affected by miscarriage (women, partners or family members), have a good understanding of English, have access to a computer with Zoom conferencing capabilities and be willing for the interview to be recorded for the purpose of transcribing the audio component at a later time.

Participants were recruited through a targeted Facebook advert which was also shared among the research teams’ existing networks including the Miscarriage Australia Facebook page (53) and the social media accounts of two pregnancy loss charity organizations Bears of Hope and Miscarriage Information Support Service (MISS). People interested in participating were asked to register their interest on a Qualtrics Plus secure survey form which commenced with the plain language statement for the study and asked a series of basic demographic and miscarriage related questions and their contact details.

Helix Qualtrics Plus is Monash University’s Australian based, HIPAA compliant instance of Qualtrics managed by the Monash Helix team. All survey data is stored securely on the Monash Helix managed data server center in Victoria, Australia. The information interested parties provided was only accessible by the research team members who have Helix approved access to Qualtrics Plus via Monash staff password protected log-in.

Interested participants were contacted by JB or AW via their preferred method (text message or email) and a suitable date and time arranged for their session. Consent was provided either verbally just prior to the session or via the signing and returning of a hardcopy consent form. Interested participants were contacted up to three times before they were deemed lost to follow up. A $50AUD digital gift voucher was provided to participants to reimburse them for their time in participating in the session.

##### Data collection

The two developers led the usability testing session with either JB or AW sitting in on most sessions to take notes. Participants could opt to attend a group or individual session. During the first usability testing session participants were asked questions relating to the mock-up and the general look and feel (branding style and tone of voice, language and terminology, information layout and imagery including icons), menu navigation options and pathways and how they would normally access the site (mobile phone or PC). In the second round of usability testing, participants were questioned about specific aspects of acceptability, usability and functionality including content, imagery, language and terminology and tone of voice. During this session they were set some basic tasks to find information on the website to test for any functionality and navigational issues. During both sessions, the think aloud method was used.

##### Data analysis

Following the usability testing, AW listened back to each session, transcribing, collating and categorizing key findings into themes related to the acceptability, usability, functionality of the website and alignment with the tone of voice guidelines. Suggested changes and areas for improvement were incorporated into the next developmental iteration to finalize the prototype. After two rounds of testing with eight participants, AW, JB and the developers met to discuss the themes. Given users’ satisfaction with website acceptability, usability, functionality and alignment with the tone of voice guidelines and recurring themes, it was decided that data saturation had been met and sufficient testing had been conducted.

## Results

### Previous study findings

The collated previous study findings relating to the online information and support needs were categorized under seven main areas including: (1) information needs—medical and emotional and other support needs, (2) when people started searching for information, (3) where people searched for information, (4) search terms used, (5) devices used, (6) what they liked about specific pregnancy loss websites, and (7) what they did not like about specific pregnancy loss websites. Under the seven main areas, relevant study findings were listed in dot point form to guide user needs and preferences and later inform content creation. At this stage, the designers read through the tabled results alongside the full published versions of the papers provided to familiarize themselves with the users and their needs and experiences of miscarriage.

### Desktop research findings

The desktop research conducted by the designers found that current pregnancy loss sites tended to fall into four categories—peer support, government sponsored websites, marketing sites or single focus sites. Peer support referred to miscarriage and pregnancy and infant loss support organizations while Government sponsored sites referred to health-related Australian Government funded websites. Marketing sites referred to those which advertised other sites miscarriage related content but not their own and single sites were those that only included one aspect of miscarriage (e.g., physical aspects only or emotional aspects only). The two top websites were Tommy’s Charity, the largest pregnancy loss support organization in Europe which funds four research centers in the UK (54) and the Miscarriage Association, a not-for-profit charity in the UK who provides information, support and advocacy for those affected by miscarriage (55).

### Stage 2

#### Affinity mapping results

The 10 interviews analyzed and synthesized as part of this study were undertaken between February 2020 and May 2020. Interviews ranged in length from 32 to 83 min. Of the 10 participants, eight were women and two were men. The mean age was 35 years (range 28–41), the mean number of miscarriages experienced by participants was 2 (range 1–3) and the mean number of living children was 1 (range 0–3). Participants lived in four of the seven states and territories in Australia.

Affinity mapping of the findings from the 10 interviews resulted in 11 overarching themes or categories, each of which contained three to five dot points relating to the theme. Table 1 outlines the 11 overarching themes resulting from the affinity mapping exercise.

**Table 1:** Eleven themes resulting from affinity mapping of user research.

Themes
1. Many women have low awareness of the symptoms of a miscarriage and the treatment needed in the event of one
2. Self-blame, loneliness and trauma are common
3. Grief can be sustained and overwhelming
4. Experiences with treatment and care are wildly divergent
5. Women’s level of access to “support networks” is extremely varied
6. Workplace experiences are mixed
7. The desire to understand “Why me?” and “How do I stop it happening again?” drives extensive research generally via Google
8. Support needs vary widely
9. Use of technology is mixed and there is no “one size fits all” in terms of preferred communication channel
10. Storytelling is a powerful relief, however there are many barriers to sharing
11. Many women feel their partners are “left out”

**Figure 2:**
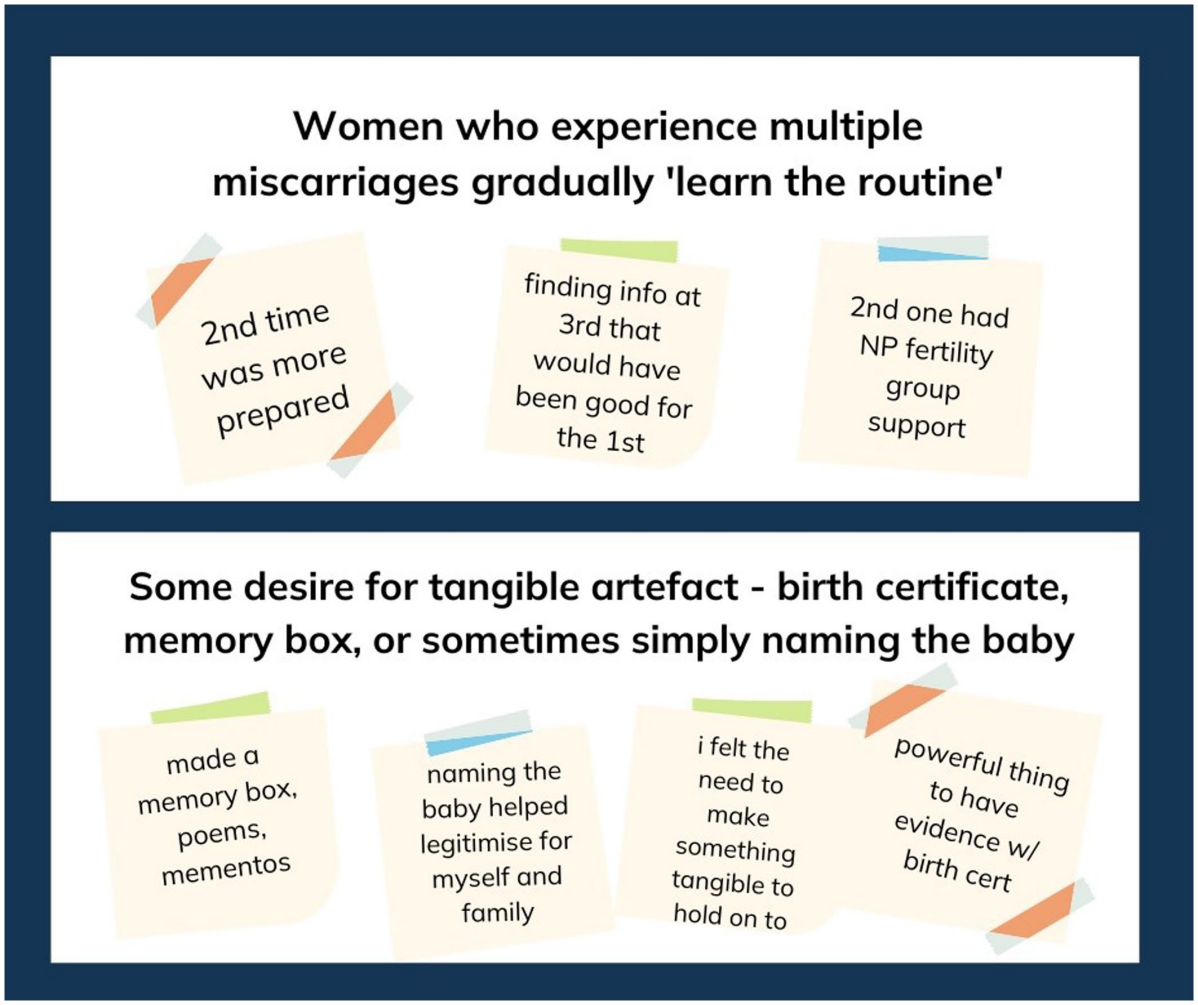
Example of post-it-note groupings of themes pertaining to the interview data.

Supplementary Appendix 1 provides the designers’ full final synthesis of the interview themes. Figure 2 provides an example of the post-it-note observations and insights grouped into emerging themes.

#### Persona results

Two personas were developed by the designers using the synthesized interview findings. Using this data, patterns in the data in terms of common experiences, goals, motivations and behaviors, were identified, allowing the designers to group similar types of users (56). Further detail was then added to personify the users, including a name, photo, scenario of their situation, their main support networks and support needs and the channels through which they are likely to seek support. This process resulted in two personas—Dalia and Ellinor.

**Figure 3:**
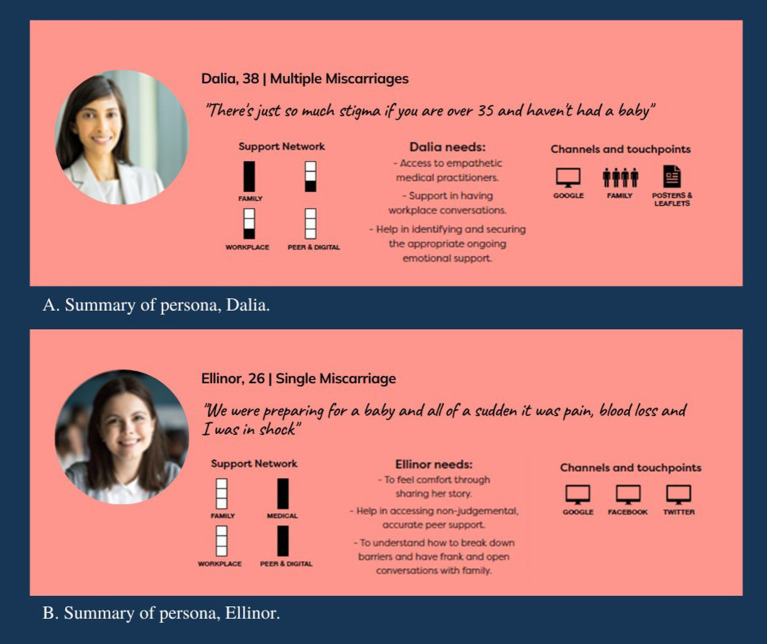
**(A)** Summary personas developed to inform the design of the Miscarriage Australia website. **(A)** Dalia persona summary. **(B)** Ellinor persona summary. Adapted with permission from Shutterstock/Michael Jung/shutterstock.com and Shutterstock/Fizkes/shutterstock.com, licensed under a Standard License.

Dalia was a 38-year-old woman who had experienced three early miscarriages. Her main support network was her family but she was struggling to find the support she and her partner needed. Her main channel for miscarriage information was Google. She needed support in having workplace conversations about miscarriage, access to empathetic medical practitioners and help in finding ongoing emotional support. She also needed reassurance that her feelings are common (see Figure 3 for persona summary and Supplementary Appendix 2 for full persona).

The other persona was Ellinor, a 26-year-old woman who had experienced a late miscarriage. She and her partner had received little support from family and friends with most coming from Ellinor’s general practitioner and her boss who had also experienced a past miscarriage. Without the support of her family and friends she turned to Google and social media for information and support but wondered if she and her partner should be feeling what they are feeling and what others do in this situation. She needed help accessing peer support and help to have frank, open conversations with her family about how she was feeling (see Figure 3 for persona summary and full personas in Supplementary Appendix 2).

### Stage 3

#### Tone of voice guideline results

**Figure 4:**
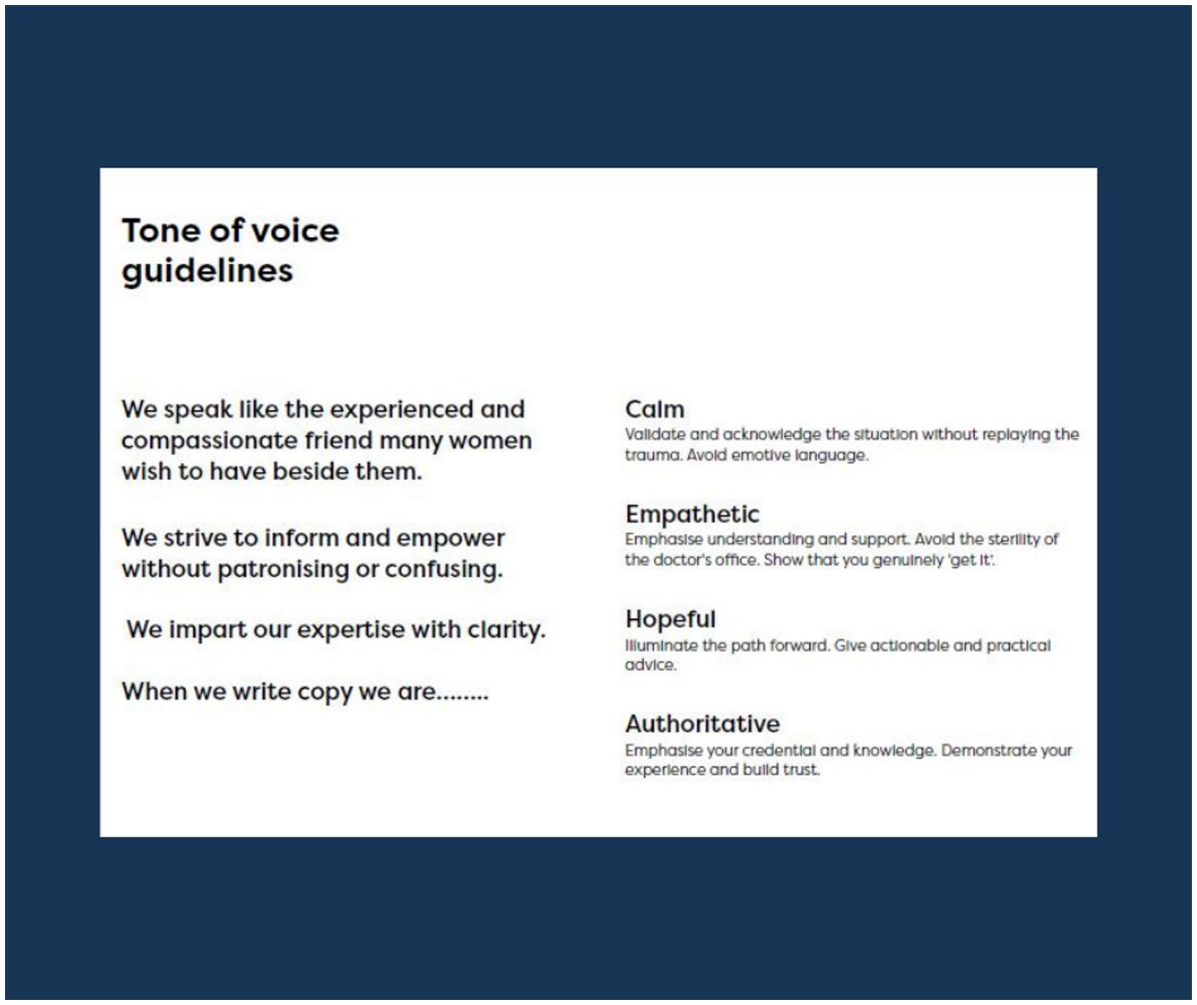
Tone of voice guidelines for Miscarriage Australia.

The personas and user research findings informed the tone of voice guidelines. Thinking about the needs of the two personas and the way Miscarriage Australia needed to meet those needs, the designers recommended that the “tone” of the website be calm, empathetic, hopeful and authoritative. In writing content for the website, the research team and designers needed to ensure the language used in the website validated and acknowledged users’ situations without replaying the trauma or using overly emotive language (calm). The website needed to show understanding and support and avoid being overly medicalized and using sterile terminology (empathetic). It needed to show the path forward, give practical, actionable advice (hopeful) and show users the team were credible, knowledgeable, experienced and trustworthy in this area (authoritative). Figure 4 shows the designers’ report related to the tone of voice guidelines.

#### Branding results

**Figure 5:**
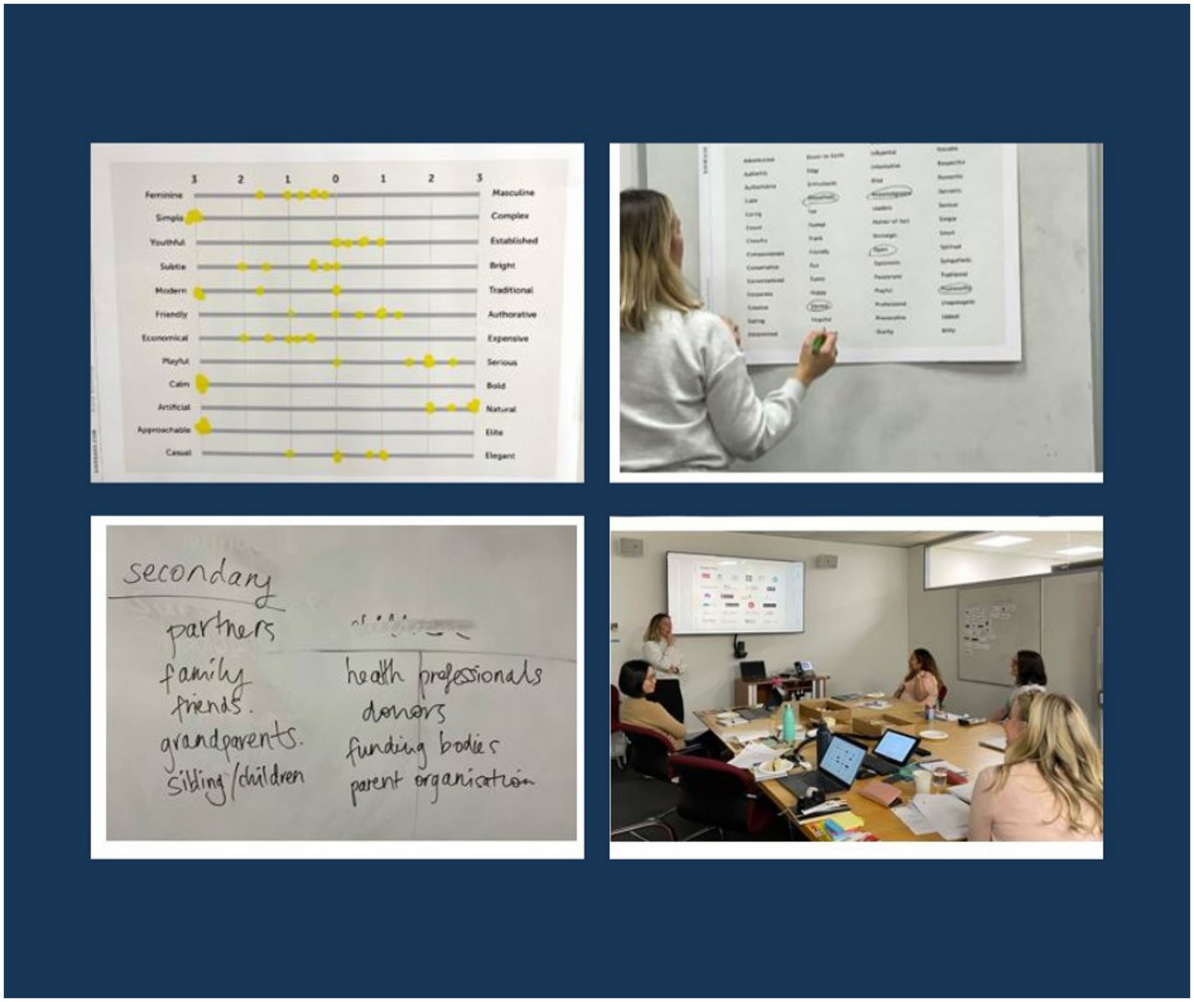
Photos of Discovery Workshop activities.

Through the Discovery Workshop the research team workshopped and identified Miscarriage Australia’s brand identity and design direction. The branding was guided by the tone of voice guidelines and the team’s knowledge of the collated previous study findings around online support needs, including what people liked and did not like about specific pregnancy websites, the synthesized interview findings and the two personas. The developers, who had expertise in human centered design, had also familiarized themselves with this information prior to the workshop. Brainstorming on a whiteboard, the team identified, discussed and reached consensus around Miscarriage Australia’s brand purpose (why we exist) and values (who we are?) which was to provide resources, support, advocacy and acknowledgement for those who have been affected by miscarriage and caring for those affected. The team identified the primary website audience as those directly affected by miscarriage (women and partners) and the secondary audience as extended family and friends, healthcare providers, donors and funding bodies. Logos of similar organizations were presented to the group for sorting into those they preferred and those they did not prefer, with reasoning discussed. The types of logos and color schemes the team preferred and felt were most appropriate for the intended audience and tone of voice, were not overly feminine in color and style (i.e., no pink or flowers), simpler, less abstract and not overly medical looking, used lower-and upper-case text and simple, not overly-cursive font (Figure 5).

**Figure 6:**
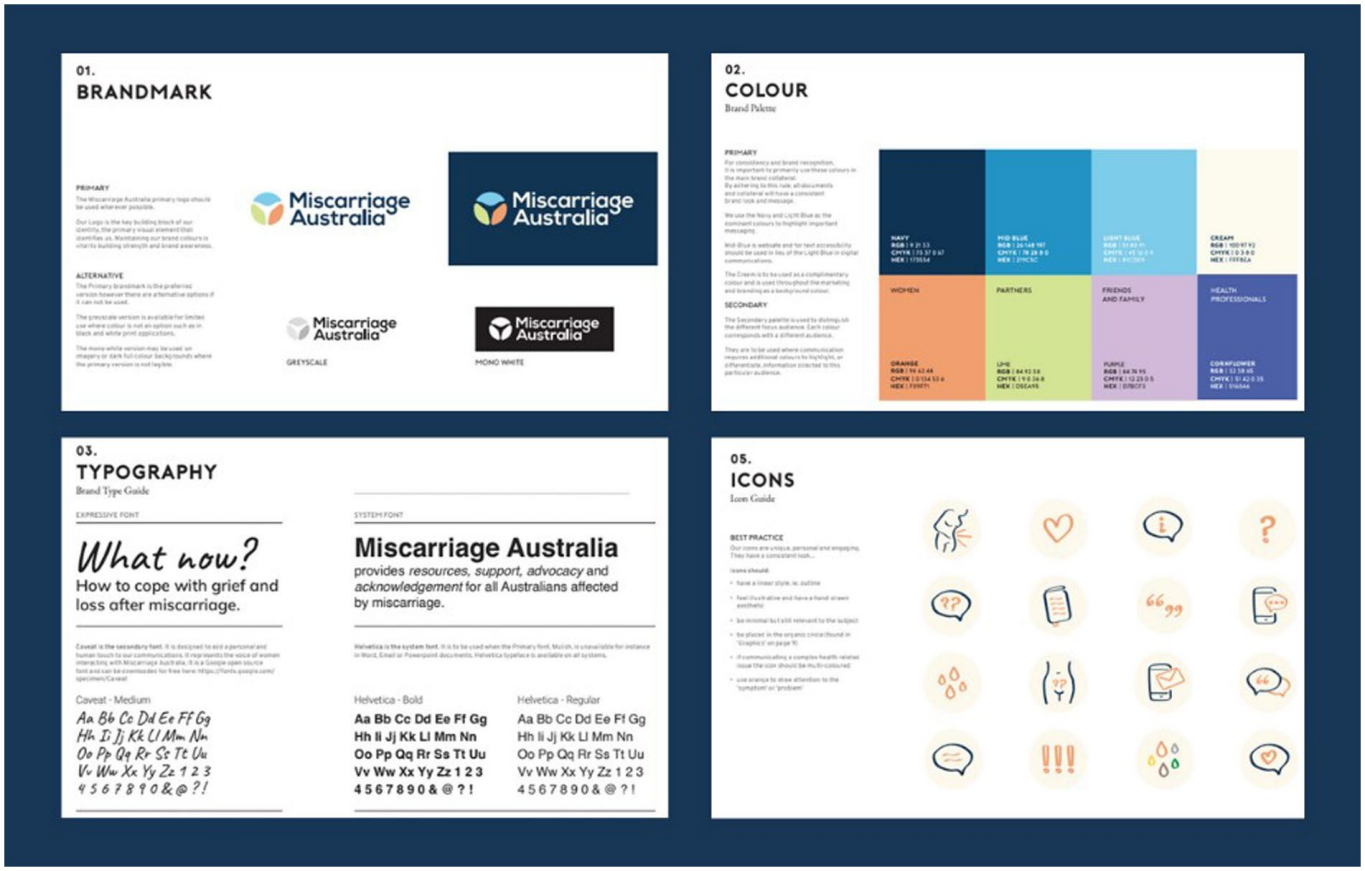
Branding elements for Miscarriage Australia.

Descriptive traits or characteristics the team felt represented Miscarriage Australia’s identity or personality were also identified on a poster, while further traits were ranked on a 7-point Likert scale by each member of the team to again guide design direction (see Figure 6). Similar to the tone of voice guidelines, the team chose words to describe Miscarriage Australia such as empathetic, honest, knowledgeable, trustworthy, open, inclusive and leaned toward a look and feel that was simple, slightly more feminine than masculine, established over youthful, subtle rather than bright, authoritative and serious, economical, calm, natural, approachable and more elegant than casual.

The final branding elements designed by the developers following the workshop and two rounds of feedback from the research team are shown in Figure 6.

#### Content creation results

**Figure 7:**
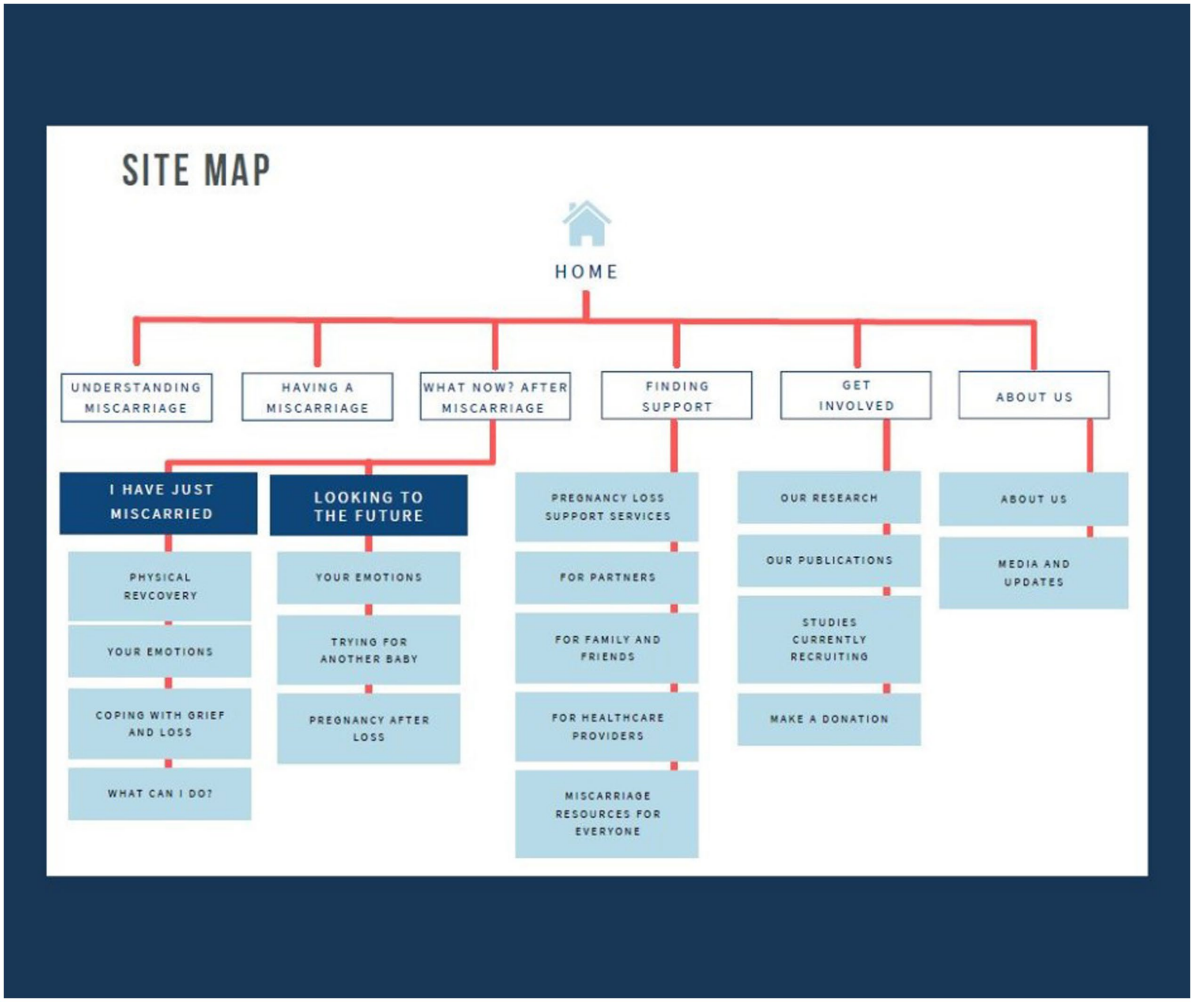
Site map for Miscarriage Australia website content creation.

Based on the user research data, a site map was developed by the research team in consultation with the designers to guide and inform content creation (see Figure 7). Over 100 pages of content was drafted and edited by the research team with relevant sections reviewed by members of the expert advisory committee. Content was informed by evidence-based academic literature in the field to ensure it was evidence based and reflected best practice. Grey literature was also referred to where appropriate and the source considered accurate and aligned with evidence-based literature and best practice in the field.

### Stage 4

#### Usability testing results

##### Participant characteristics

In total, 27 people completed an expression of interest form online. Of the 27 women, eight participated across the two rounds of testing, with two women participating in both the first and second rounds. The remaining 19 women either did not respond to further communication about the testing session or were unable to attend the scheduled session times.

Round one usability testing sessions were conducted between 26th July to 11th August 2022 while round two sessions were all conducted back to back on the 15th September 2022. Round one testing sessions ranged in time from 30 to 44 min in length. Round two testing sessions ranged from 40 to 55 min in length. Ten sessions were held in total, nine of which were single participant sessions and one was a session with two participants. All participants used a PC for the interview except one who used their mobile. Participant demographic and pregnancy related data are shown in Table 2.

**Table 2:** Participant demographic and reproductive characteristics (*n* = 8).

	Number *or* [mean and range]
Age	34 [27–43]
State	
VIC	3
NSW	3
SA	2
Ethnicity	
Australian	7
English	1
Language spoken at home	
English	7
Hindi	1
Sexuality	
Identified as heterosexual	7
Identified as bisexual	1
Education level	
Secondary school	1
TAFE diploma/certificate	1
Undergraduate university degree	4
Postgraduate university degree	2
Number of miscarriages	2 [1–4]
When experienced last miscarriage	
1–3 months ago	2
1–2 years ago	5
More than 2 years ago	1
Recruited through	
Facebook	6
Instagram	1
LinkedIn	1

#### Round one—low fidelity mockup pages results

Overall, the feedback on the low fidelity mockup pages shown in round one was very positive.

##### Acceptability and alignment with tone of voice guidelines

Most participants reported the general look and feel of the design, colors, language and terminology, typography, layout of information and imagery, including the icons, was highly acceptable. They reported the mock-up pages/website looked “very trustworthy,” “warm,” “very human,” “personable,” “reputable” and “well-informed.” As participant 3 said: *“This feels like a website where I can get information from.”* Participants described the color palette as calming, warm, soft and inviting and yet not overly feminine, noting the dark blue used throughout the website balanced some of the more feminine colors. Almost all participants felt the images on the website were appropriate and relatable except one participant who reported a couple of stock images should be replaced as the emotions portrayed by the person in the photo did not reflect the appropriate emotions related to miscarriage (e.g., a little too happy or “looks like they have a headache or just period pain rather than a miscarriage”). Two participants also noted they would like to see some imagery of objects rather than just people, particularly on the pages about remembering your baby.

Participants particularly liked the conversational font used throughout which aimed to reflect the voice of users and the quotes throughout which they felt were “very helpful,” “important” and felt as though “others were sharing their experience” with them. As participant 2 said: “*We are not alone is very reassuring … it is a comfortable thing to be greeted with*.”

The only minor terminology issues raised were that one participant felt the words “*Why me?*” on the homepage banner were a little confronting and blame inducing. Another participant felt indifferent about the use of statistics but understood others—like her partner—would like the statistics.

##### Usability

When asked about the homepage in particular, participants liked the layout and design in general, making particular note of the Miscarriage Australia mission statement, color coded tiles for different user groups (women, men, etc.), the use of statistics and thetypography.

When shown two options for the menu page, four of the six women preferred the hamburger menu option, one did not have a preference and one preferred the drop-down tab menu option. All participants reported they would likely use a mobile device to access and view the website and liked the quick exit option on the side of each page.

Functionality was tested in round two when the website prototype had been built.

#### Round two—high fidelity prototype stage results

##### Acceptability and alignment with tone of voice guidelines

Feedback from the second round of testing with the website prototype was equally positive with participants reporting the website was highly acceptable in terms of the general look and feel of the design, content, layout, language and terminology. Participants found the content easy to read and understand due to the font size, language used, terminology and layout. As participant 7 stated: “*That’s perfect. Does not feel overwhelming. It’s very clear and very easy to read*.”

When asked about specific terminology preferences, most women reported they would prefer the use of the term “baby’s remains” over more medicalized terms such as “pregnancy tissue” or “products of conception.” A couple of minor terminology issues raised were around one section heading title that was felt not to clearly convey the type of information presented in the section pages following and the use of a subsection title “Pathways of care,” the meaning of which one participant felt would not be understood unless a user was familiar with healthcare terminology.

Again, participants repeatedly described the website in line with the tone of voice guidelines—as calm, empathetic, informative, comprehensive, trustworthy and authoritative.

*The name Miscarriage Australia sounds very reputable. A big and professional organisation I can trust. (Participant 6)*

*There is a difference between this and other websites I’ve looked at. This feels a lot more like it’s been created by people that know, have experienced it and gone through [miscarriage]. While the others feel very medical and clinical …. This feels a lot more relatable. (Participant 4)*

*Miscarriage Australia comes across that is very empathetic. Comes across more approachable and supportive. (Participant 8)*

##### Usability and functionality

Overall, most participants found the website very intuitive and easy to navigate, successfully finding information on a specific topic or area when asked, for example, “Where would you go on the website to find information about support for partners?” While it was suggested the subsection title “Pathways of care” needed to be more generic, participants remarked that the information in the pathways of care page around the medical process to expect when experiencing a miscarriage, was very useful for people to know.

*I think that [the pathways of care page] is really useful. I wish I had that at the time. (Participant 8)*

*I wish I had known [about the wait times], because when I’ve gone [into the emergency department], like the worst thing in the world is happening to me … Why doesn’t anybody care? (Participant 4)*

Almost all participants commented that they wished the website had of been available when they had their miscarriage/s.

*I wish I had this [when I had my miscarriages] … It’s been a bit of a struggle … trying to find the resources and the information … trying to pull it from all different places and always Googling? and trying to find the answers is really difficult. I appreciate the fact that you are doing this. (Participant 7)*

Only minor navigational issues were discovered including most participants having difficulty finding the correct page for natural miscarriage management when directed, instead going to a page about types of miscarriage. A further issue was that most participants did not realize the main section titles on the menu page were also page links. Minor suggested changes included adding a link on each page to the “Finding support” hub page to ensure people were aware of this content from any page on the site and having an increased number of links to other pages at the bottom of each page.

## Discussion

This study reported on the design, development and evaluation of the Miscarriage Australia website using a human centered design approach. Human centered design is an approach which involves the user throughout the design process to ensure the end product or service meets their needs and requirements. Designers and developers specializing in human centered design were engaged and involved throughout the four-stage design process as were target users (those affected by miscarriage) and key stakeholders likely to refer those affected to the website (healthcare providers). Designers recommended from their analysis of user data that Miscarriage Australia’s tone of voice be calm, empathetic, hopeful and authoritative. This goal was met, with results of usability testing showing participants (users) found the design, content, imagery, language, terminology and content highly acceptable. Participants reported the website was easy to use and navigate bar a few minor functionality issues, and the content highly appropriate, in line with what they needed and the tone of voice guidelines.

The Miscarriage Australia website was developed in response to our past research. This had shown that those affected by miscarriage often struggle to find the information and support they want and need at the time of miscarriage. They wanted a reputable Australian based miscarriage website, that is accessible, comprehensive, and provides evidence-based information and advice and is informed by reputable miscarriage experts and healthcare providers (14, 30). Our past research has also shown that despite women wanting information and resources from their healthcare providers at the time of miscarriage, they often fail to receive it (31, 57), and healthcare providers often do not have ready access to it nor are they aware of the current supports services available for those affected by miscarriage (58, 59).

With more than 1 in 3 people accessing online health information in the US alone and the utilization of web-based health services more than doubling in Australia in the last decade (26, 60), the development of a web-based service such as Miscarriage Australia will likely provide a cost effective and broad reaching resource to address, in part, people’s unmet support needs. Research has shown that the utilization of eHealth and digital health services can also play a critical role in not just supporting people who are in remote areas and from low income backgrounds, but also provide a supportive tool for those who have high access to services through implementation of a stepped-care approach (61–63). Additionally, eHealth has also been shown to be beneficial in supporting the provision of care by complementing—but not replacing—the role that health professionals have in providing care (64).

Tommy’s, based in the UK, has shown that web-based information and support services can be highly utilized. Tommy’s is the largest charity in the UK providing information, support and world leading research into pregnancy and baby loss, including miscarriage. In 2021 to 2022 the Tommy’s website (54) attracted over 7.5 million website users (65). Reinforcing this, the recently updated Perinatal Society of Australia and New Zealand/Stillbirth Centre of Research Excellence guideline for perinatal bereavement care—*Guideline for Respectful and Supportive Perinatal Bereavement Care—*reported good communication as one of the five key fundamental goals of best practice perinatal bereavement care (66). As the authors noted, *“Supporting verbal information with written or electronic resources, including reliable internet sites, is widely shown to be of benefit for parents”* (66).

While using the internet, or “Dr Google” to access health information has become commonplace, it can be problematic when information is too voluminous, inaccurate or of poor quality, irrelevant or not specific to consumer’s needs, is overly jargonistic or inconsistent across multiple different sources or websites (67, 68). Concerns have also been raised by healthcare professionals that online health information may compromise or have negative effects on the consumer-healthcare provider relationship (67, 68), however, there is evidence to suggest that consumers would like healthcare providers support in navigating credible sources of online information, particularly websites (67, 69). Studies have also shown healthcare providers are supportive of patients bringing online healthcare information to them if it is accurate and relevant (68).

The Miscarriage Australia website was successfully implemented and commended as a result of using human centered design. The use of human centered design ensured users were involved throughout the design process, resulting in a comprehensive, evidence-based single source of information, that was easy to understand and navigate, and informed by reputable experts and clinicians in the field. It is a resource we anticipate healthcare professionals will feel confident and comfortable in referring patients to. With human centered design becoming an increasingly used approach for product and service design and delivery in the health space, the Miscarriage Australia website provides an ideal template or blueprint on how to develop a successful and useful digital resource for users, particularly around sensitive women’s health issues.

## Strengths and limitations

One of the main strengths of this study was the use of a human centered design approach which worked effectively in meeting user’s need for an Australian miscarriage website that was accessible, comprehensive, evidence-based and informed by reputable experts and healthcare providers. A further strength was the involvement of a broad range of multidisciplinary expert clinical advisors and a team of designers and developers who had a solid understanding and experience in human centered design principles and practices—similar to that known as the patient-centered e-health development principles (70). One of the major limitations of the study was the lack of user research around the needs of those with limited representation including LGBTIQA+ people and those with broader culturally and linguistically diverse backgrounds. The 10 interviews used to inform the personas and website development, including two male partners, were also purposively chosen to provide breadth and depth of experience. It is possible that selecting an interview sample purposively may not have provided sufficient representation of the range of miscarriage experiences and needs of individuals, in particular male partners. In stating this however, the themes generated from these interviews around miscarriage experiences and online support needs closely aligned with those from the three previous qualitative studies provided with a broader range of women and male partners. We cannot be certain however, that this was the optimal interview selection process nor sufficient to represent the range of miscarriage experiences and needs of individuals, in particular male partners. A further limitation was the lack of diversity in the participant sample for the usability testing sessions, including male partners and LGBTIQA+ individuals. It is likely a broader recruitment strategy, beyond Facebook adverts and advertising through existing networks was required to recruit a broader sample of participants. Future studies aimed at specifically exploring the online information and support needs of those with limited representation, including LGBTIQA+ people, those from culturally and linguistically diverse backgrounds and First Nations Peoples is planned. We also intend to undertake a further evaluation of the website with male partners to determine if their design and content needs differ significantly and therefore a standalone miscarriage website for men is required.

Another potential limitation of the study from a human centered or user centered design perspective is the type of online platform to meet users’ needs (a website) was already specified prior to the study commencing. While our previous research has clearly indicated the desire for an Australian based website, it is possible that there may have been another type of platform that may have also met end user’s needs. Lastly, for those who are illiterate or have no access to the internet, the website will not be useful in meeting their support needs.

## Future implications

To ensure the Miscarriage Australia website continues to meet the needs of end users, evaluation data will be collected over the following 12 months and beyond to assess website accessibility, acceptability, usefulness and impact. Users are currently invited to complete a brief online survey on the website which collects data on these aspects of use. Website use is also being monitored through Google Analytics, including collecting data on the number of people accessing the site, where they are accessing it from (i.e., country, external websites, social media links etc.), time people spend on the site, page visits and number of revisits over time. Further research is also planned to expand specific sections of the website to address the specific needs of those users with limited representation. We also intend to translate pertinent information and resources on the website into other languages to increase accessibility.

## Data availability statement

The datasets presented in this article are not readily available due to participant confidentiality and the senstive nature of the interviews. Ethics approval has not been granted to publicly share the interview transcripts that were analyzed as part of this study. Requests to access the datasets should be directed to JB, jade.bilardi@monash.edu.

## Ethics statement

Ethics approval for this study was granted on the 16th November 2020 by the Monash University Human Research Ethics Committee, Victoria, Australia (Ethics Number 25757). The participants provided their written informed consent to participate in this study.

## Author contributions

JB, V-HT, JM, GS, and MT-S contributed to the conception and design of the study. JB and AW were responsible for the interview data collation. JB and AW were responsible for writing website content while V-HT, JM, GS, and MT-S reviewed content. AW was primarily responsible for usability testing data analysis. JB was responsible for the first draft of the manuscript. All authors were responsible for critically reviewing and editing the manuscript, read, and approved the final manuscript.

## Funding

JB was in receipt of an Australian Research Council Discovery Early Career Research (Award No. DE200100049). JM was supported by the ARC Laureate Fellowship Program of Research Support (No. FL190100035).

## Conflict of interest

The authors declare that the research was conducted in the absence of any commercial or financial relationships that could be construed as a potential conflict of interest.

## Publisher’s note

All claims expressed in this article are solely those of the authors and do not necessarily represent those of their affiliated organizations, or those of the publisher, the editors and the reviewers. Any product that may be evaluated in this article, or claim that may be made by its manufacturer, is not guaranteed or endorsed by the publisher.

## Supplementary material

Supplementary Appendix 1 Synthesis of interview themes.

Supplementary Appendix 2 Full personas.
