# Supplementary material for: Miscarriage Australia: the use of a human centered design approach to design and develop a website for those affected by miscarriage
Source: Front Public Health. 2023 May 12;11:1128768. doi: 10.3389/fpubh.2023.1128768 (PMC10213628; doi:10.3389/fpubh.2023.1128768)
Supplement: Supplementary Appendix 1 — Synthesis of interview themes. [file Data_Sheet_1.PDF]

Research findings

Many women have low awareness of the symptoms of a miscarriage and the treatment needed in the event of one.

- Informed choices or even deciding what to do can be a challenge.
- Women who experience multiple miscarriages gradually 'learn the routine' through lived experience.
- There is a lack of knowledge and awareness about what to physically expect in the event of a miscarriage both at the time of miscarriage and in the weeks following (hormone changes, spotting/bleeding/heaviness leading to questions such as 'what's normal?' and 'what's going on with my body?'.
- Awareness and understanding of the physical process in the weeks following a miscarriage is a mystery to women – i.e. how their bodies respond and adjust post miscarriage. Variation of experience – the uncertainty around what their body is doing only adds to the anxiety and fear around 'something being wrong with me'.

Self-blame, loneliness and trauma are common.

- There is often a sense of failure and shame at not being able to produce a child for their partner and family, and also a sense of 'what's wrong with me?'
- The trauma of miscarriage can increase worry and stress in subsequent pregnancies.
- Women experiencing miscarriage after fertility treatment and/or after long periods attempting to conceive feel the 'weight' of what they see as lost time.
- Some women feel societal pressure to 'move on'.
- Many women are unprepared for the physical pain and trauma of MC. This makes the symptoms and the medical process all the more shocking and stressful.

Research findings

Grief can be sustained and overwhelming.

- For many couples it is the first time they have faced major grief.
- It can be a struggle to process and work through these feelings, with every experience and approach unique.
- Some couples find comfort in a tangible artefact - birth certificate, memory box, or sometimes simply naming the baby.

Experiences with treatment and care are wildly divergent.

- Some women receive excellent care.
- Others experience a purely clinical approach with a lack of emotional care and support driven by a lack of standard process and inconsistency in approach.
- Ongoing emotional support is sometimes overlooked and/or there is a lack of awareness among healthcare providers about the range of support services available.
- There is sometimes a lack of information around what to expect physically with treatment and in the weeks afterwards
- Some women leverage private health care plans to gain access to appropriate ongoing emotional support services. Lack of funds and/or an appropriate health plan is a barrier for others.

Women's level of access to 'support networks' is extremely varied.

- Some women are well supported by partners, family and friends, have friends and family who are experienced professionals, and/or have friends who have experienced miscarriage.
- Others find family and friends ill-equipped to offer support and sometimes find friends distance themselves.

Research findings

Workplace experiences are mixed.

- Some women find support in the workplace. Female leadership and/or robust employee assistance schemes can aid this.
- Others find it challenging to talk to colleagues.
- Sick leave can be a challenge when not wanting to disclose the whole story.

The desire to understand ‘Why me?’ and ‘How do I stop it happening again?’ drives extensive research generally via Google.

- Women find it hard to find informative information they trust. There's a desire for 'endorsed/trusted' information. GPs, clinicians and sites with hospital and Government affiliation seen as trustworthy.
- Women frequently want more detail in the information medical professionals provide around why the miscarriage may have occurred.
- Navigating through a wealth of information mean can be challenging. There is confronting material (such as anti-abortion sites) and questionable information (such as unmoderated chat groups).

Support needs vary widely.

- Information needs range from practical advice and where to seek help and support to nutrition, wellbeing and exercise and how to approach the subject of miscarriage with others.
- There’s a strong desire for emotional support and the need to feel heard. This is often described as an ongoing one-to-one conversation, sometimes through formal counselling and sometimes more of a ‘trusted friend’.
- Some support needs are more physical/tangible - gifts, meals, care packages.

Research findings

Use of technology is mixed and there is no ‘one size fits all’ in terms of preferred communication channel.

- Some women found online forums, Facebook etc, eased their feeling of loneliness and offered the opportunity to connect with others while retaining anonymity.
- Other women avoided social media and wished to retain a high degree of privacy.
- Views on the value of helplines, chatbots etc were also mixed.
- Some women found comfort in blogs, tele-health and fiction/novels
- ‘Traditional’ touch points such as posters and pamphlets should not be discounted.

Storytelling is a powerful relief, however there are many barriers to sharing.

- Seeking out and hearing other women's stories can break down the feeling of loneliness. This includes hearing celebrity stories.
- Telling your own story can drives connection and aids healing for some women by confronting the perceived taboo of miscarriage.
- Some women are reluctant to share experience with friends who are in different family circumstances - have children, are childless etc. Sometimes it’s simply because sharing bad news is difficult.
- Keeping pregnancy news 'secret' until 12/13 weeks causes even greater anxiety about sharing news of a miscarriage.

Many women feel their partners are 'left out'.

- Men appear reluctant to seek formal support, and those who feel comfortable in discussing miscarriage are more likely to talk to friends and family.
