# Supplementary material for: Miscarriage Australia: the use of a human centered design approach to design and develop a website for those affected by miscarriage
Source: Front Public Health. 2023 May 12;11:1128768. doi: 10.3389/fpubh.2023.1128768 (PMC10213628; doi:10.3389/fpubh.2023.1128768)
Supplement: Supplementary Appendix 2 — Full personas. [file Data_Sheet_2.PDF]

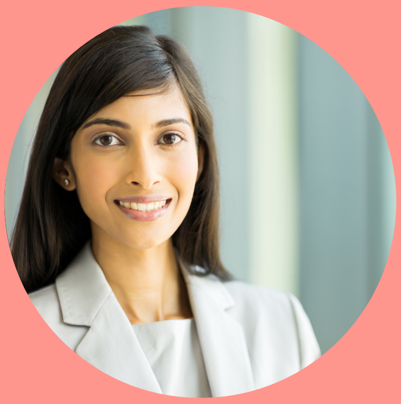

Dalia, 38

"There's so much stigma if you are over 35 and haven't had a baby."

MULTIPLE MISCARRIAGES

Support Network

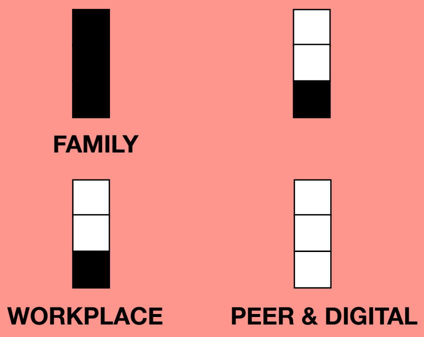

Channels and touchpoints

GOOGLE

FAMILY

POSTERS & LEAFLETS

Dalia needs:

- Access to empathetic medical practitioners.
- Support in having workplace conversations.
- Help in identifying and securing the appropriate ongoing emotional support.
- Reassurance that her feelings are common.
- Support in evaluating their next step.

About Dalia

Dalia and Saleem have been married for 12 years and are desperate to have a family. They've experienced three early term miscarriages. They have no clear answers as to why this keeps happening and are struggling to find the support they need.

Experiences with the medical profession have been desperately disappointing, with a focus on the physical aspects of miscarriage and a lack of understanding of the trauma they both feel.

Dalia wonders 'Why me?', 'Am I normal?' and 'How do I stop this happening again?'. Her need for answers has driven extensive Google searching. With each miscarriage she feels more and more grief and sadness around their losses and wonders if this is normal?

Dalia is very private. She and Saleem haven't announced the pregnancies as they've barely hit the 12-week point. They also didn't want to 'jinx' their chances of a baby.

Dalia feels under scrutiny at work. She worries that colleagues feel she takes too much sick leave. The tough times have brought her and Saleem even closer together, but Dalia worries about about what he is feeling deep down. He's trying to be strong for her but deep down Dalia thinks he may be really struggling.

They are grateful for a wonderfully supportive family and don't know what they would do without them. Despite this they have been left feeling alone and have a sense they are not coping well but don't know what to do about it.

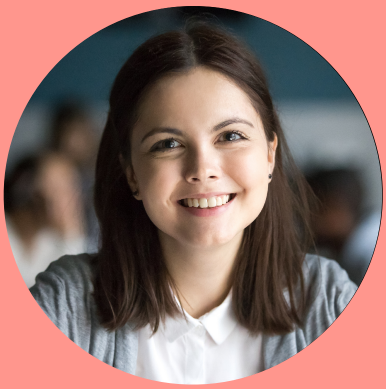

Ellinor, 26

"We were preparing for a baby and all of a sudden it was pain, blood loss and I was in shock."

SINGLE MISCARRIAGE

Support Network

FAMILY

MEDICAL

WORKPLACE

PEER & DIGITAL

Channels and touchpoints

GOOGLE

FACEBOOK

TWITTER

Ellinor needs:

- To feel comfort through sharing her story.

- Help in accessing non-judgemental, accurate peer support.

- To understand how to break down barriers and have frank and open conversations with family.

- An opportunity support others experiencing miscarriage.

About Eleanor

Pregnancy had seemed so easy for Ellinor and Thomas - other than the morning sickness - and they excitedly told friends and family their news.

They were already making plans for their first baby when they experienced a miscarriage at 15 weeks. It came as a total shock, with the added trauma of having to 'untell' their friends when they have just learnt they were pregnant.

Their family don't seem to know what to say and there are awkward silences every time they visit. Ellinor and Thomas struggle to be around friends who have children, their grief triggered by pregnancy announcements baby showers and kids birthdays.

They had the support of their GP, who is a family friend, and also from Ellinor's boss who had also experienced a miscarriage. This really helped them through the initial stages.

Ellinor needs to talk about the miscarriage and the feelings of self- blame and sadness she is feeling. Without the support of family and friends she turns to Google and social media. She finds some strange content and commentary before uncovering an extensive support network. She is now very active on a number of private Facebook groups. It feels easier to talk to strangers than to friends.

Ellinor and Thomas want to remember their baby. They plant a rose bush in their back yard to commemorate their baby but it doesn't feel like it's enough. They don't know what else they can do and question whether they are making too much of a fuss and should just be moving on. They wonder what do others do?
